# Inter-individual differences in empathy are reflected in human brain structure

**DOI:** 10.1016/j.neuroimage.2012.05.081

**Published:** 2012-09

**Authors:** Michael J. Banissy, Ryota Kanai, Vincent Walsh, Geraint Rees

**Affiliations:** a Department of Psychology, Goldsmiths, University of London, London, SE14 6NW, UK; b UCL Institute of Cognitive Neuroscience, 17 Queen Square, London, UK; c Wellcome Trust Centre for Neuroimaging, University College London, 12 Queen Square, London WC1N 3BG, UK

**Keywords:** Empathy, Voxel based morphometry, Interpersonal reactivity index, Structure, Individual differences, Social neuroscience

## Abstract

Empathy is a multi-faceted concept consisting of our ability not only to share emotions but also to exert cognitive control and perspective taking in our interactions with others. Here we examined whether inter-individual variability in different components of empathy was related to differences in brain structure assessed using voxel-based morphometry. Following a magnetic resonance imaging (MRI) scan, participants completed the Interpersonal Reactivity Index (IRI). Multiple regression was then used to assess the relationship between individual differences in grey matter volume and individual differences in empathy traits. We found that individual differences in affective empathic abilities oriented towards another person were negatively correlated with grey matter volume in the precuneus, inferior frontal gyrus, and anterior cingulate. Differences in self-oriented affective empathy were negatively correlated with grey matter volume of the somatosensory cortex, but positively correlated with volume in the insula; cognitive perspective taking abilities were positively correlated with grey matter volume of the anterior cingulate; and the ability to empathise with fictional characters was positively related to grey matter changes in the right dorsolateral prefrontal cortex. These findings are discussed in relation to neurocognitive models of empathy.

## Highlights

► We studied how individual variability in empathy is linked to brain structure. ► Affective empathy was linked to changes in the precuneus and anterior cingulate. ► Changes in the inferior frontal gyrus were also linked with affective empathy. ► Perspective taking was related to changes in the anterior cingulate. ► Personal distress was linked to changes in the somatosensory cortex and insula.

## Introduction

Empathy is a psychological construct, which describes not only our ability to share the emotions of others but also to exert cognitive control and perspective taking in our interactions. Accordingly, models of empathy highlight that the construct is multifaceted and comprises at least two key components: cognitive empathy – predicting and understanding another's mental state by using cognitive processes, and affective empathy – experiencing an appropriate emotional response as a consequence of another's state (Baron-Cohen and Wheelwright, 2004; Batson, 2009; Decety and Jackson, 2004; Preston and de Waal, 2002).

The neural mechanisms that contribute to empathy have been a topic of recent debate. Some models highlight the importance of perceptually driven activity in neural regions corresponding to observed experiences (Gallese, 2003; Goldman, 2006; Preston and de Waal, 2002). For example, simulation models of empathy contend that the observer understands the observed experience by activating similar regions of their brain that are engaged when they experience the perceived state themselves (e.g. Gallese, 2003; Goldman, 2006). Whereas, others describe empathy as an outcome of several inter-related mechanisms, including shared activations between the observer and observed, mechanisms of regulation, contextual appraisal, and mechanisms of control (Decety and Jackson, 2004; Decety and Lamm, 2006; Decety and Sommerville, 2003). For example, Decety and colleagues contend that there are three inter-related mechanisms, which give rise to cognitive (i.e. thoughts) and affective (i.e. feelings) aspects of empathy: i) shared perception–action mechanisms (Preston and de Waal, 2002), which are involved in mapping another person's experience onto the same sensorimotor and affective representations as when we experience the state ourselves; ii) regulatory mechanisms which mediate whether our empathic reactions are self (e.g. personal distress/self-oriented aversive emotional responses) or other-oriented (e.g. sympathy or compassion) (Decety and Jackson, 2006); and iii) mechanisms that aid our ability to share another's perspective (Decety and Jackson, 2004).

Functional brain imaging and transcranial magnetic stimulation studies have provided evidence in line with the notion that there are multiple mechanisms that may contribute to empathy. These include ‘shared’ neural systems in which common brain areas are activated during both self-experience and passive observation of the experiences of others (see Keysers and Gazzola, 2006; Singer, 2006 for a review); neural systems that are associated with perspective taking (Jackson et al., 2006; Ruby and Decety, 2004); and mechanisms involved in regulating distinctions between the source of one's affective response (i.e. whether our response to someone else's emotions is personal distress or is a shared feeling with the target; see Singer and Lamm (2009) for review).

The role that these mechanisms play in different aspects of empathy (e.g. cognitive, affective empathy) has also been examined. For example, lesions to sensorimotor cortices result in impairments in affective but not in cognitive empathy, whereas lesions to ventromedial prefrontal cortex result in a disruption of cognitive but not of affective empathy (Shamay-Tsoory et al., 2009). Furthermore, regions involved in affect sharing appear to be related more closely with self-oriented empathy, but can be distinguished from the neural systems that are associated with the cognitive capacity to adopt the mental states of another person (Dosch et al., 2010; Jackson et al., 2006). For example, trait levels of personal distress (measuring self-oriented aversive reactions) have been shown to positively correlate with the level of neural activity in the anterior cingulate (Cheetham et al., 2009; Lawrence et al., 2006), anterior insula (Cheetham et al., 2009), and sensorimotor cortices (Yang et al., 2009) during empathy for pain. Similarly, Jackson et al. (2006) report that self-oriented empathy towards the pain of another person (in which participants were asked to imagine observed pain/non-pain from their own perspective) leads to greater neural activity in the neural network involved in experiencing pain (e.g. secondary somatosensory cortex, anterior cingulate cortex, the insula), whereas other-oriented empathy (in which participants were asked to imagine observed pain/non-pain from another's' perspective) is associated with increases in neural activity within the inferior parietal cortex, posterior cingulate cortex, precuneus cortex, temporal–parietal junction (TPJ), and medial prefrontal cortex (also see David et al., 2006; Ruby and Decety, 2001, 2003, 2004; Vogeley et al., 2001).

In addition to personal distress, a number of other trait empathic disposition have been linked to neural activity in brain regions involved in perspective taking and sensorimotor resonance. For example, the levels of functional activation in the precuneus (Chakrabarti et al., 2006), inferior parietal cortex (Chakrabarti et al., 2006), dorsolateral prefrontal cortex (Chakrabarti et al., 2006), medial prefrontal cortex (Chakrabarti et al., 2006), inferior frontal gyrus/premotor cortex (Chakrabarti et al., 2006; Dapretto et al., 2006; Gazzola et al., 2006; Nishitani et al., 2004; Schulte-Rüther et al., 2007), somatosensory cortex (Cheng et al., 2008; Gazzola et al., 2006; Yang et al., 2009), insula (Chakrabarti et al., 2006; Jabbi et al., 2007; Lamm et al., 2011; Singer et al., 2004), anterior cingulate cortex (Decety, 2010; Singer and Lamm, 2009) and superior temporal regions (Chakrabarti et al., 2006; Schulte-Rüther et al., 2007) have all been associated with trait empathy levels.

Although functional imaging studies have examined the relationship between brain activation and empathy, few studies have sought to investigate whether underlying brain structure is related to empathy in healthy adults. Moreover, the majority of work examining measures of brain structure that contribute to empathy has focused on structural properties related to empathic deficits in a variety of pathological conditions (e.g., schizophrenia — Hooker et al., 2011; conduct disorder — Sterzer et al., 2007; frontotemporal lobar degeneration — Rankin et al., 2006). However, reverse inference from patients to healthy individuals carries a number of difficulties (Robertson and Murre, 1999), and thus the extent to which these structural differences are evident in the healthy adult brain remains an open question. To address this, here we sought to establish whether there was a relationship between trait empathy (measured using the Interpersonal Reactivity Index; Davis, 1980) and grey matter density using voxel-based morphometry (VBM) in healthy adults.

Based on previous studies highlighting the involvement of several brain regions in different facets of empathy, we expected to observe a relationship between brain structure and regions that are associated with mechanisms that support cognitive (i.e. thoughts) and affective (i.e. feelings) aspects of empathy in activation studies. For example, we expected to observe a relationship between the precuneus and perspective taking abilities (Farrer and Frith, 2002; Ochsner et al., 2004; Mar, 2011; Ruby and Decety, 2001; Vogeley et al., 2004), and between areas commonly involved in affect sharing and self-oriented empathic responses (e.g. anterior cingulate, anterior insula, inferior frontal gyrus, somatosensory cortex; Chakrabarti et al., 2006; Cheetham et al., 2009; Dapretto et al., 2006; Gazzola et al., 2006; Lawrence et al., 2006; Nishitani et al., 2004; Schulte-Rüther et al., 2007; Shamay-Tsoory et al., 2009; Yang et al., 2009).

## Methods

### Participants

One hundred and eighteen healthy participants (age mean 22.9 + 4.2 (s.d.) years old; 66 females) gave written informed consent to take part in the experiment that was approved by the local ethics committee.

### Materials and procedure

All participants completed the Interpersonal Reactivity Index (IRI; Davis, 1980); a widely used multi-dimensional measure of trait empathy, based on self-report. It consists of four subscales: Perspective taking; Personal Distress; Empathic Concern; and Fantasy (Davis, 1980; Davis et al., 1994). Empathic Concern and Personal Distress measure affective reactions but differ in their targets. Personal Distress is self-oriented and associated to aversive emotional responses in the observer (e.g. feelings of fear or discomfort at witnessing negative experiences of others). Empathic Concern is other-oriented and related to feelings of compassion and sympathy for the observed individual. Perspective Taking examines the tendency to think from another perspective (i.e. cognitive responses). Fantasy examines participants' abilities to transpose themselves into fictional situations (e.g. books, movies, daydreams).

Each subscale contained seven items. They were measured on a five point Likert scale ranging from 0 (“Does not describe me well”) to 4 (“Describes me very well”). For each subscale, a minimum score of 0 or maximum score of 28 was possible.

### MRI acquisition and analyses

High-resolution anatomical images were acquired using a T1-weighted 3-D Modified Driven Equilibrium Fourier Transform (MDEFT) sequence for each participant on a Siemens 1.5 T Sonata scanner (TR = 12.24, TE = 3.56, flip angle = 23°, field of view = 256 × 256, 176 slices, resolution = 1 × 1 × 1 mm).

The images were first segmented into grey matter and white matter using segmentation tools in Statistical Parametric Mapping software (SPM8) (http://www.fil.ion.ucl.ac.uk/spm) running on MATLAB (MathWorks, Natick, MA). Coregistration of grey matter images across participants was achieved using the DARTEL (Diffeomorphic Anatomical Registration Through Exponentiated Lie Algebra) algorithm (Ashburner, 2007). The resulting template image was transformed to MNI stereotactic space using affine and non-linear spatial normalisation with intensity modulation by the Jacobian determinant of the deformation flow field computed for each image. Then the images were smoothed with a Gaussian kernel (full-width at half-maximum, FWHM = 12 mm).

The pre-processed images were entered into a multiple regression model in SPM8 to identify cortical regions that showed a correlation with the subscales of the IRI (with all subscales included in the same design matrix). We included age, gender and total grey matter volume as covariates of no interest in the design matrix to regress out any effects attributable to them. The inclusion of gender as a covariate of no interest was of particular importance, as sex differences have been reported for all subscales of the IRI (Davis, 1980). In line with previous studies (e.g. Rankin et al., 2006), our decision to focus our analysis on IRI subscales rather than total IRI score, was because some components of the IRI have been shown to negatively correlate with social competence (e.g. Personal Distress scale; Davis, 1983).

We conducted region of interest analyses using previous functional brain imaging studies of empathy to constrain our anatomical hypotheses. In particular, we focused our analysis on the anterior cingulate (MNI coordinates: ± x = 3, y = 24, z = 33; Singer et al., 2004), inferior frontal gyrus (x = 60, y = 14, z = 24; Lamm et al., 2011), precuneus (± x = 10, y = − 50, z = 36; Mar, 2011), anterior insula (± x = 39, y = 9, z = − 21; Singer et al., 2004), somatosensory cortex (± x = 48, y = − 16, z = 54; Hooker et al., 2008), and dorsolateral prefrontal cortex (± x = 42, y = 39, z = 24; Lamm et al., 2011). These regions were selected based on previous functional brain imaging studies demonstrating their involvement in affective and cognitive empathy tasks, and meta-analyses of brain regions involved in affect sharing and metalizing. In situations where regions of interest were reported in more than one study we choose the coordinate from the study with a higher number of citations. Statistical significance was assessed using small volume correction (Worsley et al., 1996) at a threshold of P < 0.05 (corrected) for those clusters that also passed a whole-brain uncorrected threshold of P < 0.001 within a sphere (10 mm radius) centred at each of the coordinates identified by our prior hypotheses. Outside these pre-defined regions, we used a statistical threshold of P < 0.05 corrected for the whole-brain volume at a cluster level using non-stationary correction (Hayasaka et al., 2004).

## Results

### IRI scores

Scores on each subscale of the IRI were consistent with previously published norms for this measure (Davis, 1980; Table 1). Previous findings have also reported sex differences on each subscale of the IRI (Davis, 1980), with females displaying higher scores than males in each case. We observed a similar pattern of results (Table 1), with females scoring higher on the Empathic Concern scale [t(116) = 2.55, P = .012] and the Personal Distress scale [t(95.23) = 2.93, P = .004]. There was also a trend for females to score higher than males on the Fantasy scale, t(116) = 1.55, P = .123. We were unable to replicate differences between male and female scores on the Perspective Taking subscale, t(116) = .438, P = .662, however it is of note that this represents the smallest identified sex difference on all four subscales of the IRI (Davis, 1980).

In addition, scores on the Fantasy scale were positively correlated with scores on the Perspective Taking (R = .307, P = .001) and Empathic Concern (R = .224, P = .015) scales. Scores on Perspective Taking were also positively correlated with scores on Empathic Concern (R = .464, P = <.001). No other correlations were found across the subscales.

### Region of interest structural analysis

Each region-of-interest was defined according to our prior hypotheses (see Methods) and statistical correction undertaken for the small volume examined. This analysis revealed that inter-individual variability on the Fantasy scale showed a significant positive correlation with brain volume in the right dorsolateral prefrontal cortex (R = 0.29, T(110) = 3.15, P_FWE_corr_ = 0.012; peak MNI coordinate within the s.v.c. sphere x = 40, y = 47, z = 30). No other regions of interest showed a significant correlation with the scale of Fantasy (P > 0.05, corrected; Table 2).

Inter-individual variability in participants' Empathic Concern negatively correlated with grey matter volume in the left precuneus (R = − 0.27, T(110) = 2.89, P_FWE_corr_ = 0.023; peak MNI coordinate within the s.v.c. sphere x = − 8, y = − 49, z = 45); and in the left anterior cingulate (R = − 0.25, T(110) = 2.69, P_FWE_corr_ = 0.037; peak MNI coordinate within the s.v.c. sphere x = − 4, y = 27, z = 28). We also found a significant negative relationship with Empathic Concern at our ROI on the anterior insula. The peak coordinate was found within the inferior frontal gyrus (R = − 0.35, T(110) = 3.98, P_FWE_corr_ = 0.001; peak MNI coordinate within the s.v.c. sphere x = − 48, y = 6, z = 18), but this is consistent with the cluster from the original study used to define the ROI extending to this region. No other regions of interest showed a significant correlation with the scale of Empathic Concern (P > 0.05, corrected; Table 2).

Inter-individual variability in Perspective Taking showed a significant positive correlation with grey matter volume of the left anterior cingulate (R = 0.25, T(110) = 2.69, P_FWE_corr_ = 0.037; the coordinate of the peak x = − 6 y = 33, z = 31). No other regions of interest including precuneus, which showed a significant correlation with empathic concern, showed a significant correlation with the Perspective Taking scale (P > 0.05, corrected; Table 2).

As for Personal Distress, we found a significant positive correlation with grey matter volume of the left anterior insula (R = 0.27, T(110) = 2.92, P_FWE_corr_ = 0.022; the coordinate of the peak x = − 32, y = 9, z = − 18). We also found a negative correlation with the grey matter volume of the somatosensory cortex (R = − 0.32, T(110) = 3.57, P_FWE_corr_ = 0.004; the coordinate of the peak x = − 45, y = − 19, z = 61). No other regions of interest showed a significant correlation with the Personal Distress scale (P > 0.05, corrected; Table 2).

### Whole brain analysis

At a threshold of P < .05 corrected for the whole-brain volume at a cluster level using non-stationary correction, we found a significant negative relationship between scores on the Empathic Concern subscale and grey matter volume in the left inferior frontal gyrus (R = − 0.36, T = 4.06, P = < 0.05, corrected for multiple comparisons across the whole brain volume; the coordinate of the peak x = − 51, y = 8, z = 18). We did not observe any other regions at this corrected threshold.

## Discussion

This study examined whether individual differences in trait empathy dimensions were related to morphological differences in human brain structure. Our results suggest that inter-individual variability in different aspects of empathy was linked with distinct morphological changes in separate brain regions. Affective empathic abilities that are oriented towards another person (i.e. Empathic Concern subscale) were linked with reduced grey matter volume within the left precuneus, left inferior frontal gyrus, and left anterior cingulate; a tendency towards self-oriented affective empathy was linked with reduced grey matter volume in the left somatosensory cortex, but increased volume in the left insula; cognitive perspective taking abilities were linked to increased grey matter volume in the left anterior cingulate; and the ability to empathise with/place oneself into fictional situations (Fantasy subscale) was associated with increased grey matter volume in the right dorsolateral prefrontal cortex.

To our knowledge, our study is the first to examine the relationship between regional grey matter volume and different facets of empathy in healthy adults. Our findings show that the grey matter structure in brain regions implicated in previous studies of functional commonalities in empathy can also account for inter-individual variability in empathic traits. For example, the involvement of the precuneus cortex, insula, anterior cingulate and dorsolateral prefrontal cortex is consistent with recent meta-analyses highlighting these regions as components of core networks involved in affect sharing (Lamm et al., 2011) and perspective taking (Mar, 2011). The relationship between changes in brain structure of the inferior frontal gyrus and empathic abilities is consistent with findings showing a relationship between trait empathy and functional levels of neural activity in this brain region (Chakrabarti et al., 2006; Gazzola et al., 2006; Hooker et al., 2008, 2010; Jabbi et al., 2007), and neuropsychological patient data showing that lesions to the inferior frontal gyrus result in impairments in affective empathy (Rankin et al., 2006; Shamay-Tsoory et al., 2009). Finally, the association with changes in the somatosensory cortex and levels of personal distress is in line with growing evidence pointing to a role for somatosensation in social perception (Banissy et al., 2010, 2011; Hooker et al., 2008, 2010; Keysers et al., 2010; Pitcher et al., 2008).

The relationship between structural variations in each brain region and specific empathy traits is also interesting. For example, our finding that decreased brain volume in the inferior frontal gyrus was associated with increased scores on the Empathic Concern subscale of the IRI is consistent with previous work examining the neural correlates of dispositional measures of empathy. The Empathic Concern subscale of the IRI is other-oriented and related to affective empathy. In healthy adults, functional neural activity in the inferior frontal gyrus has been linked to trait levels of empathic concern in a number of studies (e.g. Schulte-Rüther et al., 2007). Previous findings have also indicated, that lesions to the inferior frontal gyrus result in impairments in affective but not in cognitive empathy (Shamay-Tsoory et al., 2009) and damage to the inferior frontal gyrus has been linked to scores on the empathic concern subscale of the IRI (Rankin et al., 2006). Our evidence that reduced brain volume in the inferior frontal gyrus was related to increased levels of empathic concern is therefore consistent with this data. The specific direction of the relationship also implies that in healthy adults “less is more”. While this may first seem paradoxical to the typical “more is better” notion, such a relationship is not uncommon (e.g. see Jung et al., 2010; Kanai et al., 2011) and may reflect differences in maturation during adolescence and cortical thinning. Moreover, it has been postulated that cortical thinning during maturation reflects changes in functional activation during skill acquisition, with plasticity decreasing as efficiency increases (Durston and Casey, 2006; Durston et al., 2006). In this context, less grey matter volume in the inferior frontal gyrus showing a relationship with increased empathic concern scores would seem consistent with the patient data and functional imaging studies described above.

The trade off between increased local grey matter volume in the anterior cingulate being related to increased scores of the Perspective Taking subscale of the IRI, but decreased grey matter volume in the anterior cingulate being associated with increased scores on the Empathic Concern subscale of the IRI is also intriguing. A recent meta-analysis of empathy for pain highlights the anterior cingulate as part of a core network in this process (Lamm et al., 2011). In relation to trait levels of empathy, previous functional brain imaging studies have associated levels of neural activity in the anterior cingulate with trait levels of perspective taking (measured on IRI) (Montag et al., 2008) and personal distress (measured on IRI) (Cheetham et al., 2009; Lawrence et al., 2006). Our data relating brain volume in the anterior cingulate to perspective taking abilities is therefore consistent with some of the studies of functional correlates of empathy. They extend them by indicating that the anterior cingulate may also be important in other empathic capacities including more affective empathic dispositions that are other-oriented. Precisely why less grey matter volume in the anterior cingulate may facilitate affective empathy (i.e. empathic concern), but more grey matter volume in the same brain region may facilitate cognitive perspective taking is difficult to disentangle. This is because the links between macroscopic volumetric measures such as regional grey matter volume and functional differences are barely understood. The idea of cortical thinning and maturation, postulated above (in the case of empathic concern), is one of many possible explanations. To reach a full understanding of this issue, it will be important to investigate how differences in microstructure measures (e.g., the number of neurons, the degree of myelination, the degree of dendritic arborization and so on) are associated with functional differences and how they contribute to differences in the aggregate measure of grey matter volume across tasks.

We found a negative relationship between scores on the Personal Distress subscale of the IRI and structural brain changes in the somatosensory cortex. Recent findings have implicated a prominent role for the somatosensory system in social perception, with studies indicating that somatosensory-related cortices play a key in role in using social cues to understand mental and emotional states of others (e.g. Banissy et al., 2010, 2011; Hooker et al., 2008, 2010; Keysers et al., 2010; Pitcher et al., 2008). Previous findings have shown that scores on the Personal Distress subscale of the IRI are negatively correlated with social competence and social perception abilities (Davis, 1983). In this regard, a negative relationship between scores on the Personal Distress subscale and brain volume in the somatosensory system may indicate that less grey matter volume in the somatosensory system is related to poorer social perception abilities and is therefore consistent with prior studies that highlight the role of somatosensation in our ability to use social cues to infer emotions and mental states. In contrast, the positive relationship between brain volume in the anterior insula and personal distress implies that more brain volume in the anterior insula is negative for social perception abilities.

Our findings are also interesting in a broader context of the neurobiology of individual differences. A number of recent functional neuroimaging studies have begun to go beyond considering functional activation that is common to all subjects, to examine predictive links between brain activity and individual trait-behaviours (see Hariri, 2009; Kanai and Rees, 2011 for review). This has led to a variety of developments in our understanding of how trait-like behaviours modulate variability in brain function (e.g. trait anxiety modulates amygdala reactivity to affective stimuli — Dickie and Armony, 2008; ventral striatum responsiveness is associated with individual differences in impulsivity — Hariri et al., 2006) and to the suggestion that differential patterns of brain activity may act as markers for individual differences in personality and liability for disease (Hariri, 2009). While a limited number of studies have examined the influence of trait empathy on functional brain activity, none have considered the relationship between structural variation and inter-individual differences in trait empathy in healthy adults. Our findings therefore provide novel predictive links between trait-empathy and region-specific structural variation. They indicate that brain regions previously identified in studies of functional commonalities in the neuroscience of empathy are susceptible to individual variation in brain structure and that this contributes to inter-individual differences in specific components of trait-empathy. An important next step will be to determine the mechanisms driving this variability and to consider the extent to which individual variation in these components may provide predictive markers for vulnerability towards social perception deficits. In this context, it is also important to consider the extent to which inter-individual differences in empathy are a consequence of, or contribute to, the structural differences that we observe. Moreover, while levels of trait empathy are enduring and show continuity across time and situations from early childhood (Volbrecht et al., 2007; Zahn-Waxler et al., 1992, 2001), the relative contributions of environmental and biological factors in the development of empathic abilities have been a topic of much interest (e.g. Knafo et al., 2008; Volbrecht et al., 2007; Zahn-Waxler et al., 2001). Our findings add a new dimension to this by implicating specific intra-individual differences in brain structure that may determine empathic cognition or vice versa.

Taken together, we examined how individual variability in different components of empathy related to volumetric differences in brain structure. Our findings demonstrate that different aspects of empathy are linked with distinct morphological changes in separate brain regions and suggest that multiple mechanisms are associated with increases in specific empathic skills. This implies that empathy is multi-faceted and that structural variation in brain regions that support affect sharing and cognitive perspective taking acts to facilitate self and other related empathic processes in different ways.

## Figures and Tables

**Table 1:** Scores on each IRI subscale for all subjects, males and females (mean ± standard deviation).

IRI subscale	All participants	Male participants	Female participants
Fantasy	16.47 ± 4.12	15.86 ± 3.80	16.98 ± 4.31
Perspective taking	18.45 ± 3.95	18.29 ± 3.83	18.59 ± 4.06
Personal distress	11.83 ± 4.66	10.43 ± 5.07	12.94 ± 4
Empathic concern	20.09 ± 4.68	18.76 ± 4.41	21.05 ± 4.69

**Table 2:** Region of interest structural analysis (P = <.05 corrected using small volume correction) examining cortical regions related to scores on each IRI subscale with all subscales included in the same design matrix. For each region, from left to right, we describe: the anatomical description of the region of interest; the IRI subscales that correlated with it; the R value; the MNI coordinates of the peak coordinate with the s.v.c sphere; and the corrected P value.

Anatomical location	IRI subscale	R value	MNI coordinates			P value
x	y	z
Left precuneus	Empathic concern	− 0.27	− 8	− 49	45	0.023
Left anterior cingulate	Empathic concern	− 0.25	− 4	27	28	0.037
Perspective taking	0.23	− 6	33	31	0.037
Left somatosensory cortex	Personal distress	− 0.32	− 45	− 19	61	0.004
Left insula	Personal distress	0.27	− 32	9	− 18	0.022
Empathic concern	− 0.35	− 48	6	18	0.001
Right dorsolateral prefrontal cortex	Fantasy	0.29	40	47	30	0.012
